# Personal Care Product Use Predicts Urinary Concentrations of Some Phthalate Monoesters

**DOI:** 10.1289/ehp.8083

**Published:** 2005-07-18

**Authors:** Susan M. Duty, Robin M. Ackerman, Antonia M. Calafat, Russ Hauser

**Affiliations:** 1 Department of Environmental Health, Occupational Health Program, Harvard School of Public Health, Boston, Massachusetts, USA; 2 Department of Nursing, School for Health Studies, Simmons College, Boston, Massachusetts, USA; 3 National Center for Environmental Health, Centers for Disease Control and Prevention, Atlanta, Georgia, USA; 4 Vincent Memorial Obstetrics and Gynecology Service, Andrology Laboratory and In Vitro Fertilization Unit, Massachusetts General Hospital, Boston, Massachusetts, USA

**Keywords:** environment, personal care products, phthalates, urinary metabolites

## Abstract

Phthalates are multifunctional chemicals used in a variety of applications, including personal care products. The present study explored the relationship between patterns of personal care product use and urinary levels of several phthalate metabolites. Subjects include 406 men who participated in an ongoing semen quality study at the Massachusetts General Hospital Andrology Laboratory between January 2000 and February 2003. A nurse-administered questionnaire was used to determine use of personal care products, including cologne, aftershave, lotions, hair products, and deodorants. Phthalate monoester concentrations were measured in a single spot urine sample by isotope dilution–high-performance liquid chromatography coupled to tandem mass spectrometry. Men who used cologne or aftershave within 48 hr before urine collection had higher median levels of monoethyl phthalate (MEP) (265 and 266 ng/mL, respectively) than those who did not use cologne or aftershave (108 and 133 ng/mL, respectively). For each additional type of product used, MEP increased 33% (95% confidence interval, 14–53%). The use of lotion was associated with lower urinary levels of monobutyl phthalate (MBP) (14.9 ng/mL), monobenzyl phthalate (MBzP) (6.1 ng/mL), and mono(2-ethylhexyl) phthalate (MEHP) (4.4 ng/mL) compared with men who did not use lotion (MBP, 16.8 ng/mL; MBzP, 8.6 ng/mL; MEHP, 7.2 ng/mL). The identification of personal care products as contributors to phthalate body burden is an important step in exposure characterization. Further work in this area is needed to identify other predictors of phthalate exposure.

Phthalates are used industrially as plasticizers and solvents and as stabilizers for colors and fragrances. They are found in personal care products, medications, paints, adhesives, and medical equipment made with polyvinyl chloride plastics [Agency for Toxic Substances and Disease Registry (ATSDR) 1995, 2001, 2003]. Diethyl phthalate (DEP), di(2-ethylhexyl) phthalate (DEHP), butylbenzyl phthalate (BBzP), and di-*n*-butyl phthalate (DBP) are used in personal care products (Houlihan et al. 2002; Koo and Lee 2004). The potential effects of phthalates on human health are not well characterized. There is a paucity of existing data describing phthalate-associated human health outcomes, although animal studies have found testicular toxicity associated with phthalate exposure (Li et al. 1998; Parks et al. 2000).

Two studies provide preliminary evidence of associations between urinary concentrations of monoethyl phthalate (MEP), a metabolite of DEP, and DNA damage in human sperm (Duty et al. 2003a), as well as relationships of monobutyl phthalate (MBP) and monobenzyl phthalate (MBzP) phthalate, metabolites of DBP and BBzP, respectively, with decreased sperm motility (Duty et al. 2003b). In a recent epidemiologic study prenatal exposure to MEP, MBP, MBzP, and monoisobutyl phthalate was associated with shortened anogenital distance (AGD) in male infants (Swan et al. 2005). In rodent studies AGD is a sensitive measure of prenatal antiandrogen exposure.

Despite the recent public and scientific interest on the potential human health effects of phthalates, routes of human exposure to phthalates have not been adequately characterized. Potential routes include dietary ingestion of phthalate-containing foods, inhalation of indoor and outdoor air, and dermal exposure through the use of personal care products that contain phthalates. As far as we know, the proportional contribution of phthalate-containing personal care products to total body burden has not been studied. Houlihan et al. (2002) quantified phthalate levels in 72 personal care products obtained at a supermarket in the United States, including hair gel/hair spray, body lotion, fragrances, and deodorant. DEP was detected in 71% of these products, DBP in 8%, BBzP in 6%, and DEHP in 4% of the products tested (Houlihan et al. 2002). In a recent study (Koo and Lee 2004), high-performance liquid chromatography (HPLC) was used to quantify the levels of the same four phthalates in 102 hair sprays, perfumes, deodorants, and nail polishes purchased at retail stores in Seoul, Korea. DBP was detected in 19 of the 21 nail polishes and in 11 of the 42 perfumes; DEP was detected in 24 of the 42 perfumes and 2 of the 8 deodorants.

The assertion that phthalates are absorbed into the circulation through human skin is physiologically plausible and is supported by a limited number of human and animal studies (ATSDR 1995, 2001, 2003). The stratum corneum of the epidermis regulates transdermal absorption, and uptake is achieved through passive diffusion (Howard et al. 2001). Water-soluble substances penetrate hydrolyzed keratin, whereas lipid-soluble substances such as phthalates, especially DEP and other low-molecular-weight phthalates, can dissolve into lipid materials between keratin filaments. After penetration of the epidermis, diffusion into the dermal and subcutaneous layers is generally uninhibited because of the nonselective and porous aqueous mediums in these layers. Substances can then enter the systemic circulation through venous and lymphatic capillaries. With increased hydration, rates of absorption of more hydrophilic compounds can be increased 3–5 times more than usual. Epidermal permeability also varies greatly between species (Howard et al. 2001).

In one study dermal doses of DEHP were administered to human volunteers over a 24-hr period, and approximately 1.8% of the total dose was absorbed (Wester et al. 1998). Another experiment involved the topical application of DBP to human volunteers. The authors determined that 68 mg would be absorbed in 1 hr if the skin surface of the whole body were saturated with the chemical (Hagedorn-Leweke and Lippold 1995). In another study human breast skin was exposed *in vitro* to ^14^C-DEP, and average absorption under conditions of occlusion was 3.9% compared with 4.8% without occlusion at 72 hr. However, this was much slower and less complete compared with absorption through rat skin (Mint et al. 1994).

*In vitro* and animal experiments have also indicated that phthalates are absorbed percutaneously (Barber et al. 1992; Deisinger et al. 1998; Elsisi et al. 1989; Melnick et al. 1987; Mint et al. 1994; Ng et al. 1992; Scott et al. 1987). However, the mechanism explaining differential rates of uptake is not agreed upon. Scott et al. (1987) attributed the phthalate-specific rates of absorption to varying degrees of lipophilicity. Elsisi et al. (1989) observed that the lengths of the alkyl chains were inversely associated with the relative rates of absorption; except for dimethyl phthalate, DEP has the shortest alkyl chain (ATSDR 1995).

Although diester and monoester phthalates have short biologic half-lives of approximately 6–12 hr and do not accumulate (ATSDR 1995, 2001, 2003), the frequent application of personal care products may result in semi-steady-state levels, making it possible to estimate typical phthalate body burden from a single urine sample (Hauser et al. 2004; Hoppin et al. 2002). After exposure, diester phthalates, which may be found in personal care products, are metabolized to monoester metabolites, the suspected toxic agents (Li et al. 1998). For this reason and to avoid contamination, monoester phthalate metabolites rather than the parent diesters are commonly measured (Blount et al. 2000).

Our objective in the present study was to determine whether the use of personal care products predicted urinary levels of phthalate monoesters, and to identify subject characteristics that predicted phthalate levels.

## Materials and Methods

### Design and setting.

This study was approved by the Human Subject Committees at the Harvard School of Public Health, Massachusetts General Hospital (MGH), and Simmons College. All subjects signed an informed consent. Subjects were participants in an ongoing study on phthalates and male reproductive health. They were recruited between January 2000 and February 2003 from the Andrology Laboratory at MGH. Males between 20 and 54 years of age who were partners of subfertile couples were eligible; those who have had a vasectomy were excluded. Approximately 65% of eligible men agreed to participate. The most frequently cited reason for not participating was lack of time. A total of 406 men were recruited.

### Personal care product use assessment.

A trained research nurse administered a brief questionnaire to each subject at the time of his visit to the MGH andrology clinic for semen and urine sample collection. Information was obtained on personal care product use, smoking status, age, height, weight, race, and use of medications. Participants were specifically asked whether they had used hair gel/hair spray, lotion, aftershave, cologne, or deodorant in the 48 hr before the collection of the urine sample. They were also asked to record the time they last used the products within the 48-hr period.

### Urinary phthalate monoester measurement.

A single spot urine sample was collected from each participant in a sterile plastic specimen cup (which was prescreened for phthalates) on the same day that the questionnaire was administered. The analytical approach has been described in detail (Blount et al. 2000) and adapted to both enable the detection of additional monoesters and improve efficiency of the analysis (Silva et al. 2003). Briefly, measurement of monoester metabolites, namely, MEP, MBP, mono(2-ethylhexyl) phthalate (MEHP), MBzP, and monomethyl phthalate (MMP), entailed enzymatic deconjugation of the phthalates from their glucuronidated form, solid-phase extraction, HPLC separation, and tandem mass spectrometry detection. The limits of detection (LODs) were approximately 1 ng/mL. One method blank, two quality control samples (human urine spiked with phthalate monoesters), and two sets of standards were analyzed along with every 21 unknown urine samples. Analysts at the Centers for Disease Control and Prevention (CDC) were blind to all information concerning subjects. To control for urinary dilution, urinary concentrations were adjusted according to specific gravity. Specific gravity was measured using a handheld refractometer (National Instrument Company Inc., Baltimore, MD). The following formula was used to adjust phthalate concentrations by specific gravity: *P_c_* = *P*[(1.024 − 1)/SG − 1], where *P_c_* represents specific gravity–corrected phthalate concentration (ng/mL), *P* is the measured phthalate concentration (ng/mL), and SG is the specific gravity of the sample. Specific gravity–adjusted monoester phthalate levels were used as continuous outcome variables in statistical models.

### Statistical methods.

All analyses were performed using SAS software (version 8.1; SAS Institute Inc., Cary, NC). The use of each personal care product was categorized into a dichotomous variable (yes/no use in the 48 hr before the urine sample collection).

Because the phthalate monoester levels were not normally distributed, nonparametric tests were used to assess univariate associations between personal care product use and urinary phthalate levels. Multiple linear regression was used to explore the relationship between each of the five personal care products and each of the five log-transformed monoester phthalate concentrations. In addition, a six-level sum variable was created, representing the number of different types of products used by a participant in the past 48 hr; possible values for this variable were 0, 1, 2, 3, 4, or 5. To determine if a dose–response relationship existed between urinary phthalate levels and the number of types of personal care products used, a trend test was performed using sum variable as an ordinal variable. For urinary phthalate concentrations that were below the LOD, a value equal to half the LOD was imputed (except when quantification was given) as follows: MEP, 0.605 ng/mL; MBzP, 0.235 ng/mL; MBP, 0.47 ng/mL; MEHP, 0.435 ng/mL; and MMP, 0.355 ng/mL.

After evaluating appropriateness using quadratic terms, we modeled age and body mass index (BMI; kilogram per square meter) as continuous independent variables. Smoking status was categorized as current smoker and current nonsmoker (includes ex-smokers and never smokers). Race was coded as African American, Hispanic, and other race, with Caucasian as the reference group. On the basis of biologic plausibility and statistical factors (i.e., change in parameter estimate), we included age, BMI, race, and smoking variables in all models as potential confounders.

To explore the relationship between time of product use and urinary levels of the phthalates, we regressed log-phthalate levels on the time between product use and urine sample collection (referred to as TIMEDIF). TIMEDIF was categorized into four intervals: product use 0–3 hr before urine sample collection (TIME0–3); > 3 but ≤6 hr (TIME3–6); > 6 but ≤8 hr (TIME6–8), and > 8 hr (TIME9). Approximately 75–85% of subject’s product use was within 8 hr of urine collection, and therefore we used TIMEDIF > 8 hr as the reference category.

## Results

### Subject demographics.

Of the 406 men recruited for an ongoing semen quality study, 37 did not provide urine samples. Of the remaining 369, specific-gravity analyses were not available for 32, leaving 338 for primary analysis. Additionally, one urine sample was missing MMP concentrations. The study population was composed largely of white (*n* = 275, 82%), nonsmoking men (*n* = 304, 91%) (Table 1). There were 19 African-American men, 18 Hispanic men, and 24 men of other race/ethnicity.

### Personal care product use.

Eleven men (3%) did not provide complete product use information (Table 1). Most men reported use of deodorant (89%), whereas fewer men reported using hair gel (37%), lotion (34%), cologne (29%), and aftershave (13%). Nine men (2.7%) did not use any of the personal care products listed on the questionnaire, 114 (33.7%) used only one type of product, 119 (35.3%) used two types of products, 71 (21%) used three types of products, 22 (6.5%) used four different types of products, and only 3 (0.9%) of the men used five or more different types of products within 48 hr of urine collection. The percentage of African-American (59%) and Hispanic (53%) men who reported using cologne within 48 hr of urine collection was higher the percentage of Caucasian men (25%) or men of other races (25%). Additionally, African-American men (65%) were more likely than Hispanic (44%), Caucasian (30%), or men of other races (43%) to use lotion. No other associations were seen between any other personal care products and race. Interestingly, men who used aftershave were almost twice as likely (18.5%) to also use cologne as non-aftershave users (9.8%) (chi squared *p* = 0.03). There were no consistent relationships among any of the other products used.

### Urinary phthalate monoesters.

There was a wide distribution of both specific gravity–adjusted (Table 2) and -unadjusted phthalate monoester levels (Table 3). Five phthalate monoesters were detected in 75–100% of subjects. MEP was the most prevalent (100%), followed by MBP (95%) and MBzP (90%). MEHP and MMP were both found in about 75% of subjects. Phthalate metabolite concentrations are presented both adjusted for specific gravity and unadjusted for comparison with other studies. The highest geometric mean levels were found for MEP (179 ng/mL), followed by MBP (16.6 ng/mL), MBzP (7.1 ng/mL), MEHP (6.6 ng/mL), and last, MMP (4.5 ng/mL).

### Covariate relationships.

Race and cigarette smoking status were predictors of MEP and MBP levels (Table 4). We found significantly higher median MEP levels among African-American men (506 ng/mL) and Hispanic men (395 ng/mL) compared with Caucasian men (140 ng/mL) and those men categorized as other race (125 ng/mL). Median MBP levels in Caucasian men (15.3 ng/mL) were also lower than among African-American men (32.7 ng/mL) and Hispanic men (29.1 ng/mL), and among men identified as other race (26.5 ng/mL). Median MEP levels in current smokers (250 ng/mL) were significantly higher than among nonsmokers (143 ng/mL) (Table 4). BMI was weakly, although positively, correlated with MEP (Spearman correlation coefficient of 0.1, *p* < 0.05). Age was not associated with any of the five phthalate concentrations. Wilcoxon rank-sum tests showed positive associations between the sum variable for product use and African-American men and men of other races compared with Caucasians. BMI was positively associated with those identified as other race.

### Product use and urinary phthalate relationship.

**Figure 1:**
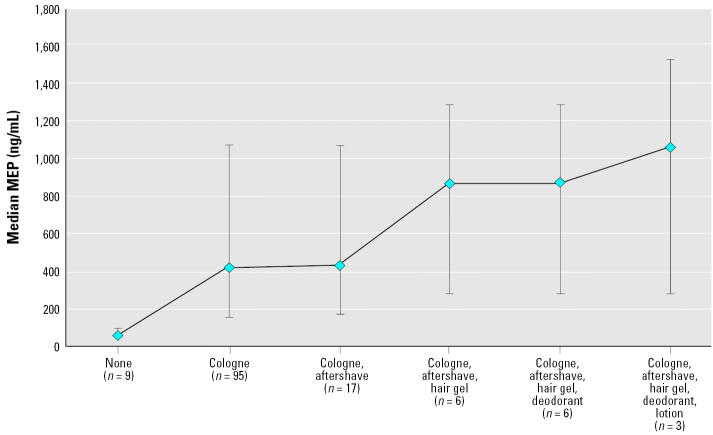
Specific-gravity–adjusted urinary MEP concentration according to combinations of product types used. Data points represent medians; error bars represent 25th and 75th percentiles.

In the univariate analyses, median MEP levels were higher among cologne users (265 ng/mL) compared with those who did not use cologne (108 ng/mL). Likewise, men who used aftershave had higher median MEP levels (266 ng/mL) than men who did not (133 ng/mL). Fragranced products such as cologne and aftershave contain relatively higher DEP levels than other personal care products. Figure 1, created on a subset of men who used cologne plus additional products, depicts the rise in MEP levels with specific combinations of personal care product use.

Median MBP was lower among men who had used deodorant (16.3 ng/mL) compared with those who did not use deodorant (22.5 ng/mL). The use of lotion was associated with lower median levels of MBP (14.9 ng/mL), MBzP (6.1 ng/mL), and MEHP (4.4 ng/mL) compared with men who did not use lotion (MBP, 16.8 ng/mL; MBzP, 8.6 ng/mL; MEHP, 7.2 ng/mL) (Table 5).

Men of Hispanic, Caucasian, and other races who used cologne had considerably higher median MEP levels (981, 444, and 178 ng/mL, respectively) than non-cologne users of similar race (138, 102, and 116 ng/mL; *p* = 0.09, < 0.001, and 0.13, respectively). Interestingly, African-American men who used cologne had 30% lower median MEP levels compared with non-cologne users (371 ng/mL vs. 508 ng/mL), although the differences were not statistically significant. Hispanic and Caucasian men had substantially higher MEP levels if they used aftershave (1076 and 220 ng/mL) than if they did not (138 and 126 ng/mL; *p* = 0.08 and 0.03, respectively). African-American men who used aftershave had 33% lower MEP levels compared with non-aftershave users (340 ng/mL vs. 508 ng/mL), although the differences were not statistically significant. No other race/product associations were observed.

### Multiple linear regression.

**Figure 2:**
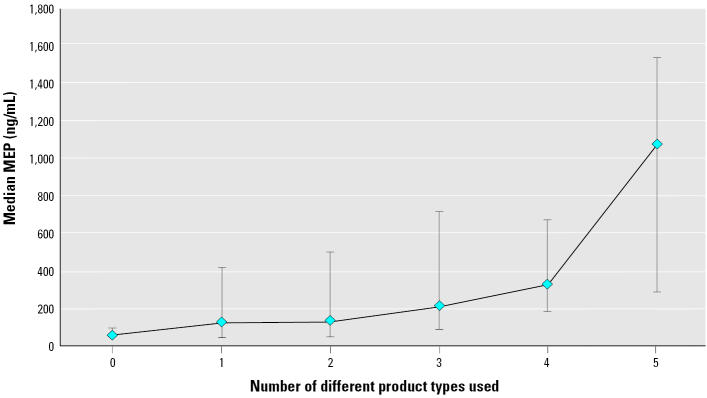
Specific-gravity–adjusted urinary MEP concentrations according to number of product types used. Data points represent medians; error bars represent 25th and 75th percentiles.

In multiple linear regression models, after adjusting for race, smoking status, BMI, and age, urinary levels of MEP were 2.57 times higher among men who had used cologne and 1.71 times higher among aftershave users compared with men who did not report the use of these products (*p* < 0.0001 and 0.02, respectively) (Table 6). There was also a dose–response relationship between urinary phthalate MEP levels and the number of types of personal care products used. For every additional type of product used, MEP concentrations increased 33% (95% confidence interval, 14–53%; trend test *p* = 0.0002) (Figure 2). The use of deodorant was associated with 30% lower MBP levels (*p* = 0.08). MBP, MBzP, and MEHP levels were 31% (*p* = 0.004), 34% (*p* = 0.003), and 34% (*p* = 0.003) lower, respectively, among men who had used lotion within the past 48 hr before urine collection compared with men who had not.

### Time of product use.

In secondary analyses, we explored the relationship between time of product use and urinary levels of phthalate monoesters. Statistical power was limited in these secondary analyses as a result of small sample sizes, generally fewer than 15 subjects for each of the four TIME strata. The analyses were performed only among users of each specific product. Cologne use at TIME0–3, TIME3–6, and TIME6–8 compared with cologne use at TIME9 was associated with an increase in MEP of 1.7-fold (*p* = 0.17), 2.8-fold (*p* < 0.01), and 1.1-fold (*p* = 0.75), respectively. No consistent time trends were observed for the other phthalates and cologne use. Aftershave was inconsistently associated with a 2- to 3-fold increase in MEP levels—3.0-fold increase at TIME0–3 (*p* = 0.15), 2.0-fold increase at TIME3–6 (*p* = 0.25), and 2.6-fold increase at TIME6–8 (*p* = 0.11)—compared with aftershave use at TIME9. No time trends were observed for the other phthalates and aftershave use. For lotion use at TIME0–3, TIME3–6, and TIME6–8, MBP concentration increased 1.9-fold (*p* = 0.03), 1.2-fold (*p* = 0.55), and 1.2-fold (*p* = 0.52) compared with lotion use at TIME9. No significant time relationships were found between lotion use and any other phthalate or between deodorant or hair gel use and any of the phthalates.

## Discussion

In the present study, men who used cologne and/or aftershave within the 48-hr period before the collection of the urine sample had higher urinary levels of MEP. This is not unexpected because previous studies have demonstrated that DEP, the parent compound of MEP, is an ingredient in many colognes, deodorants, and fragranced products (Houlihan et al. 2002; Koo and Lee 2004) and that percutaneous absorption of DEP occurs (Api 2001; ATSDR 1995; Mint et al. 1994; Scott et al. 1987). More striking is the steepness of the dose–response relationship between the number of product types used in the 48 hr before urine collection and urinary MEP levels. DEP was found in 71% of the personal care products tested in one study (Houlihan et al. 2002), whereas DEHP, DBP, and BBzP were found in fewer than 10% of products. In another study, DEP was found in 57% of the perfumes and 25% of the deodorants surveyed; DBP, DEHP, and BBzP were not detected in any of the deodorants and in fewer than 27% of the perfumes (Koo and Lee 2004). Therefore, it is plausible that MEP would have a strong relationship with multiple product use, whereas the other phthalate monoesters would not.

Interestingly, the use of body lotion was associated with lower levels of MBP, MBzP, and MEHP. The reason for this relationship is not known, although several hypotheses are plausible. It is possible that other ingredients in body lotion may act as a barrier to the absorption of DBP, BBzP, and DEHP. It is also feasible that men who use lotion use fewer other personal care products. However, chi-squared tests did not show significant inverse relationships between the use of body lotion and other products (data not shown). An alternative explanation is that the urinary levels of these monoesters reflect exposure to their parent phthalates other than by use of personal care products. Percutaneous absorption after dermal exposure is expected to be lower for DBP, BBzP, and DEHP than for DEP.

The quantities of phthalates present in different brands of deodorant, aftershave, hair gel/hair spray, lotion, and cologne are known to be quite variable (Houlihan et al. 2002; Koo and Lee 2004). In the present study, because information on the use of specific brand name products was not gathered, the analysis was performed by category of product. This approach is likely to introduce bias toward the null because not all products within a given category contain phthalates and those that do contain phthalates do so at variable concentrations. Because the participants in this study are all male, it is unclear whether the findings of this study may be generalizable to women, who may use different types and combinations of personal care products.

It is unclear why current smokers had higher levels of MEP. The results, however, were unstable because the sample size was small: only 31 men (9%) were current smokers. One potential explanation is that smoking may alter the toxicokinetics of DEP. Although DBP, unlike DEP, is listed as an ingredient in the filters of Phillip Morris cigarettes (Phillip Morris 2004), MBP was not found to be related to current smoking status.

Racial differences in urinary levels of MEP and MBP were consistent with previous data from the National Health and Nutrition Examination Survey (NHANES) 1999–2000 that have shown African Americans and Hispanics have higher urinary levels of MEP and MBP than do Caucasians (CDC 2003; Silva et al. 2004). In our study we explored the MEP and race associations for use of specific personal care products. The higher MEP levels for Hispanic than for Caucasian men appeared related to differentially higher cologne and aftershave use. Interestingly, the higher urinary MEP levels in African-American than in Caucasian men did not appear to be related to higher cologne and/or aftershave use. Therefore, the use of other products not identified in this study, different sources of exposure to DEP, or differential toxicokinetics may be driving the high MEP levels among African-American men. After accounting for race, age, and smoking status in the statistical models, MEP levels were still significantly higher among cologne and aftershave users; African-American race remained an independent predictor of MEP levels. However, it is important to note that only 18 African Americans participated in the study, and these findings may be related to chance because of the small numbers. Further study on racial/ethnic differences is warranted.

In an earlier study on the relationship between demographic characteristics and urinary phthalate levels among a nonrepresentative subset of 289 participants of NHANES III, MBP, MBzP, and MEHP were higher in individuals of low socioeconomic status (Koo et al. 2002). Urban residence was also significantly associated with higher MEHP and MBP levels. Socioeconomic status and area of residence were not controlled for in the present study, and these factors could potentially account for some of the differences measured between the racial groups. Finally, it is also possible that higher personal care product use or the selection of certain types of products among racial groups may contribute to differences in urinary phthalate levels.

The time elapsed between product use and urine sample collection influenced the relationship between cologne use and MEP concentrations. MEP was 2.7-fold higher when cologne was used between 3 and 6 hr before urine collection compared with when it was used 8 hr or more before urine collection. Therefore, to best assess the relationship of cologne use on urinary MEP levels, we suggest that urine collection should occur 3–6 hr after cologne use.

When time of lotion use was not accounted for in the analysis, there was an inverse association between urinary levels of MBP and lotion use. However, in analyses in which time of use was explored, MBP concentrations were significantly higher within the first 3 hr after lotion use compared with lotion use 8 hr or more before. The lotion use MBP relationship may require a larger data set to determine how use correlates with MBP levels in urine samples collected at variable times after applying lotion.

Although aftershave use between 0 and 8 hr before urine collection was associated with 2- to 3-fold higher MEP concentration compared with aftershave use more than 8 hr before urine collection, each strata had fewer than 10 subjects, and the reference group had only 15. This could explain why the aftershave–time of use relationships did not reach statistical significance.

To put these findings into perspective, a comparison with previous work is offered. The interquartile difference (443 ng/mL) in MEP, associated with increased DNA damage in sperm (Duty et al. 2003b), was approximately 2- to 3-fold higher than the difference in levels of MEP observed between men who did versus those who did not use cologne (312 ng/mL) or aftershave (131 ng/mL), respectively. MBP and MBzP, found in our previous study to be associated with decreased sperm motility and concentration (Duty et al. 2003b), were not found to be associated with aftershave or cologne use.

## Conclusions

Cologne and aftershave use were associated with significantly higher urinary MEP levels after controlling for age, BMI, smoking, and race. Additionally, a dose–response relationship was found between the number of different types of personal care products used and MEP urinary concentrations. Interestingly, lotion was inversely associated with most phthalate levels. Secondary analysis revealed that, for cologne, product use 3 to 6 hr before urine collection was most predictive of urinary MEP concentration. However, for lotion, product use in the 3 hr before urine collection was most predictive for MBP concentration.

The identification of personal care products as contributors to phthalate body burden is an important step in exposure characterization. Additionally, the results of this study suggest that the time that products are used in relation to the time that the urinary samples are collected should be documented. This will help reduce random measurement error in statistical analysis. Further work is needed to identify additional predictors of phthalate exposure.

## Figures and Tables

**Table 1:** Characteristics of study subjects (*n* = 338).

Characteristic	Value
Age [median (25%, 75%)]	35.0 (32.0, 39.1)
BMI [median (25%, 75%)]	27.5 (25.0, 30.6)
Race^a^ [*n* (%)]	
White	275 (82)
Black/African American	18 (5)
Hispanic	19 (6)
Other	24 (7)
Smoking^b^ [*n* (%)]	
Current smoker	31 (9)
Nonsmoker (ex- and never smoker)	304 (91)
Use of personal care products [*n* (%)]	
Lotion^c^	110 (34)
Hair gel/hair spray^d^	121 (37)
Aftershave^e^	42 (13)
Deodorant^f^	299 (89)
Cologne^g^	94 (29)

a Race data missing for 2 men.

b Smoking data missing for 3 men.

c Lotion use data missing for 11 men.

d Hair gel/hair spray data missing for 7 men.

e Aftershave data missing for 8 men.

f Deodorant data missing for 1 man.

g Cologne data missing for 8 men.

**Table 2:** Distribution of specific gravity–adjusted urinary levels of phthalate monoesters: percentiles and summary statistics (ng/mL).

		Percentile						
Phthalate	*n*	5th	25th	50th	75th	95th	Mean ± SD	Geometric mean
MEP	338	24.5	58.2	154	503	2,030	490 ± 979	179
MEHP	338	< LOD	2.4	6.3	19.1	116.1	27.6 ± 69.1	6.6
MBP	338	3.1	10.3	16.5	30.6	68.2	76.2 ± 798.4	16.6
MBzP	338	< LOD	4.0	7.7	14.1	39.7	14.0 ± 34.6	7.1
MMP	337	< LOD	2.1	4.8	11.4	32.1	10.8 ± 22.8	4.5

LODs (ng/mL): MEP, 1.21; MBzP, 0.47; MBP, 0.94; MEHP, 0.87; MMP, 0.71.

**Table 3:** Distribution of unadjusted urinary levels of phthalate monoesters: percentiles and summary statistics (ng/mL).

		Percentile						
Phthalate	*n*	5th	25th	50th	75th	95th	Mean ± SD	Geometric mean
MEP	338	17.0	48.5	145	457	1,953	485 ± 1,008	164
MEHP	338	< LOD	1.9	5.2	18.4	134.6	25.6 ± 60.1	6.0
MBP	338	2.2	7.8	14.5	31.7	75.1	85.6 ± 932.8	14.9
MBzP	338	< LOD	2.9	6.8	14.1	41.3	13.90 ± 32.4	6.0
MMP	337	< LOD	1.7	4.5	10.1	31.3	11.0 ± 31.6	4.1

LODs (ng/mL): MEP, 1.21; MBzP, 0.47; MBP, 0.94; MEHP, 0.87; MMP, 0.71.

**Table 4:** Median (25th and 75th percentiles) urinary levels of phthalate monoesters (ng/mL)^a^ by race and smoking status.

	MEP	MEHP	MBP	MBzP	MMP
Race					
Black	506* (294, 1,134)	7.4 (3.9, 8.7)	32.7* (18.1, 42.5)	10.7 (6.8, 21.4)	6.0 (2.0, 12.0)
Hispanic	395* (83.3, 1,076)	5.6 (3.3, 20.7)	29.1* (17.3, 42.4)	12.2 (3.5, 19.5)	5.2 (1.9, 11.9)
Other	125 (40.3, 218)	7.1 (1.7, 10.8)	26.5* (7.1, 38.4)	6.4 (2.3, 11.9)	4.6 (2.0, 11.3)
White	140 (56.6, 469)	6.2 (2.3, 20.7)	15.3 (9.9, 26.9)	7.4 (4.0, 14.2)	7.3 (3.3, 11.3)
Smoking					
Yes	250* (96.5, 826)	5.0 (1.8, 12.6)	20.9 (10.8, 46.9)	8.0 (4.3, 17.4)	8.5 (4.6, 18.2)
No	144 (57.5, 465)	6.4 (2.5, 19.6)	16.3 (10.3, 30.0)	7.7 (4.0, 14.2)	4.5 (2.1, 10.4)

a Specific gravity–adjusted phthalate levels.

*Univariate regression analysis *p* ≤0.05; reference group for race is whites.

**Table 5:** Median (25th and 75th percentiles) urinary levels of phthalate monoesters (ng/mL)^a^ by personal care products used 48 hr before urine sample collection.

	MEP	MEHP	MBP	MBzP	MMP
Lotion					
Yes	136 (60.0, 438)	4.6* (1.6, 11.5)	15.7* (8.5, 28.1)	6.1* (3.0, 11.5)	4.2 (2.0, 10.4)
No	160 (56.6, 528)	7.2 (2.7, 20.4)	16.8 (10.3, 31.7)	8.6 (4.6, 15.1)	5.1 (2.4, 12.1)
Cologne					
Yes	422* (155, 1076)	5.3 (1.8, 16.9)	18.6 (12.2, 33.6)	10.5 (4.6, 16.7)	4.7 (2.6, 13.2)
No	110 (48.0, 293)	6.6 (2.5, 19.1)	15.4 (9.6, 29.2)	6.8 (3.9, 13.8)	4.9 (2.1, 10.3)
Deodorant					
Yes	165 (60.4, 534)	6.1 (2.4, 20.7)	16.3 (10.3, 29.2)	7.7 (4.0, 14.2)	4.7 (2.0, 11.3)
No	91.4 (36.4, 323)	6.3 (2.2, 13.7)	22.5 (10.8, 39.0)	7.5 (4.9, 14.0)	6.9 (2.6, 12.6)
Aftershave					
Yes	266* (123.2, 625)	6.1 (2.7, 13.8)	17.9 (10.9, 33.6)	7.9 (4.2, 16.2)	4.8 (2.4, 8.6)
No	135 (54.5, 477)	6.3 (2.3, 18.9)	16.3 (10.2, 30.2)	7.6 (3.9, 14.1)	4.8 (2.1, 12.0)
Hair gel/spray					
Yes	182 (57.8, 547)	7.7 (2.6, 21.6)	16.0 (8.9, 24.5)	8.0 (4.2, 14.2)	4.9 (2.0, 11.4)
No	139 (58.2, 464)	5.9 (2.1, 14.6)	16.7 (11.1, 31.7)	7.6 (4.0, 14.2)	4.8 (2.3, 11.7)

a Specific-gravity–adjusted phthalate levels.

*p ≤0.05 in multivariate linear regression models adjusted for age, BMI, race, and smoking.

**Table 6:** Multiplicative factors^a^ (95% confidence interval) for a change in urinary phthalate monoester level^b^ associated with use of personal care products within the past 48 hr (*n* = 323).

Product type	MEP	MEHP	MBP	MBzP	MMP
Lotion	0.97 (0.70–1.33)	0.66 (0.44–0.99)	0.69 (0.53–0.88)	0.66 (0.50–0.87)	0.92 (0.66–1.29)
Cologne	2.57 (1.88–3.53)	0.96 (0.63–1.46)	1.06 (0.82–1.38)	1.16 (0.88–1.54)	1.19 (0.84–1.67)
Deodorant	1.24 (0.75–2.05)	1.23 (0.65–2.34)	0.70 (0.47–1.04)	0.95 (0.62–1.47)	0.94 (0.55–1.59)
Aftershave	1.71 (1.10–2.64)	0.93 (0.53–1.64)	1.00 (0.70–1.43)	1.16 (0.80–1.69)	0.97 (0.61–1.55)
Hair gel	1.15 (0.85–1.57)	1.23 (0.83–1.81)	0.92 (0.72–1.17)	0.95 (0.73–1.24)	0.99 (0.72–1.35)

a All models are adjusted for age, BMI, smoking, and race. Multiplicative factors represent multiplicative changes in phthalate levels associated with use of specific personal care products within the past 48 hr after back-transformation of phthalate concentrations: 1.0, no change in urinary phthalate level; < 1.0, multiplicative decrease in phthalate level; > 1.0, multiplicative increase in phthalate level.

b In all models, log transformations of specific gravity–adjusted phthalate concentrations were used.
